# Study on the Influence of Hydroxyapatite on Human Cell Viability and Adhesion in Chemical Antibacterial Silver Coatings

**DOI:** 10.3390/dj14040202

**Published:** 2026-04-01

**Authors:** Vlad-Gabriel Vasilescu, Andreia Cucuruz, Lucian Toma Ciocan, Miruna S. Stan, Florin Miculescu, Ionela Cristina Voinea, Cosmin Mihai Cotruț, Andreea Veronica Dediu-Botezatu, Elisabeta Vasilescu, Ana Maria Țâncu, Marina Imre, Silviu Mirel Pițuru

**Affiliations:** 1 Discipline of Dental Prosthesis Technology, Faculty of Dentistry, Carol Davila University of Medicine and Pharmacy, Dionisie Lupu Street, No. 37, District 2, 020021 Bucharest, Romania; vlad.vasilescu@umfcd.ro; 2 Biomaterials and Medical Devices Department, Faculty of Medical Engineering, National University of Science and Technology Politehnica Bucharest, 1-7 Gh. Polizu Street, District 1, 011061 Bucharest, Romania; andreia.cucuruz@upb.ro; 3 Department of Biochemistry and Molecular Biology, Faculty of Biology, University of Bucharest, 91-95 Splaiul Independentei, 050095 Bucharest, Romania; ionela-cristina.voinea@bio.unibuc.ro; 4 Faculty of Materials Science and Engineering, National University of Science and Technology Politehnica Bucharest, 313 Splaiul Independenței, J Building, 060042 Bucharest, Romania; florin.miculescu@upb.ro (F.M.); cosmin.cotrut@upb.ro (C.M.C.); 5 Department of Chemistry, Physics and Environment, Faculty of Science and Environment, Dunărea de Jos University, Domnească Street, 111, 800201 Galați, Romania; andreea.botezatu@ugal.ro; 6 General Association of Engineers in Romania (AGIR), Victoriei Boulevard 118, 030167 Bucharest, Romania; elisabeta.vasilescu@yahoo.com; 7 Discipline of Prosthodontics, Faculty of Dentistry, Carol Davila University of Medicine and Pharmacy, Dionisie Lupu Street, No. 37, District 2, 020021 Bucharest, Romania; anamaria.tancu@umfcd.ro (A.M.Ț.); marina.imre@umfcd.ro (M.I.); 8 Department of Organization, Professional Legislation and Management of the Dental Office, Faculty of Dental Medicine, Carol Davila University of Medicine and Pharmacy, 17-23 Plevnei Street, 020021 Bucharest, Romania; silviu.pituru@umfcd.ro

**Keywords:** TiZr bioalloy, silver (Ag), antibacterial effect, cytotoxicity, hydroxyapatite (HAp), biocompatibility, cell viability, human osteoblasts

## Abstract

**Objectives**: In dental implantology, the priorities in scientific research are to identify solutions that guarantee a beneficial biomaterial–tissue interaction, both in terms of implant biointegration and protection against infections. The experimental approach consisted of chemical deposition of silver (Ag), silver and hydroxyapatite (HAP) on a TiZr metallic support. The aim of the research is to study the influence of hydroxyapatite on the possible adverse effects produced by silver in antibacterial coatings. **Methods**: The characterization of the coating was performed by scanning electron microscopy (SEM) and EDS spectroscopy, XRD diffraction and FT-IR infrared analysis. In vitro cell viability and adhesion testing was performed by quantitative (MTT) and qualitative fluorescence-based assays on samples (without deposition and with chemical deposition), in the presence of human fetal osteoblasts (hFOB cell line) after 8 days of incubation. **Results**: The findings of the study indicate an increase in cell viability by combining silver with hydroxyapatite. Preliminary data indicated a cell viability of 20% when the metal support is coated exclusively with silver and 60% in the presence of hydroxyapatite in the silver coating. **Conclusions**: The experimental study offers insights into the potential cytotoxic effects of silver in antibacterial coatings. Co-deposition with hydroxyapatite improved osteoblast viability compared to surfaces coated with silver alone, indicating that it may have a beneficial effect in Ag-based surface functionalization. The underlying mechanism (e.g., modulation of silver species/ion release) was not directly quantified in this work and should be addressed in future studies.

## 1. Introduction

Oral implantology is a viable solution for treating totally or partially edentulous patients, but whose clinical efficacy and long-term success require ensuring a beneficial biomaterial–tissue interaction.

The use of biomaterials with high biocompatibility and the creation of biomedical interfaces with biocompatible properties through various techniques are determining factors that make the implant–tissue assembly become an integrated, stable and durable system [1,2,3,4,5,6,7].

However, there is an important category of factors that contribute decisively to the success of the dental implant insertion process, respectively, surgical factors and those related to the avoidance of infections that may occur in adjacent tissues, as a result of defective handling. This contamination is usually organic and exhibits high levels of low-oxygen carbon and bacterial contamination during surgical placement of the dental implant [8,9,10,11,12].

From this point of view, it is known that implanted biomaterials are particularly susceptible to microbial colonization and capable of favoring the appearance of infections. Gristina (1987) pointed out that a major disadvantage of implanted devices remains “the possibility of bacterial adhesion to biomaterials, which causes biomaterial-centric infections and lack of successful tissue integration, or compatibility with biomaterial surfaces” [13].

This type of contamination can occur when bacteria that are naturally present in the oral tissues colonize the subgingival surface of the implant and affect the healing process and implicitly the osseointegration process [14,15,16].

Bacterial adhesion to dental implants (the presence of biofilms on the surface of the implant) can cause peri-implant diseases, such as peri-implant mucositis (inflammation of the soft tissue around dental implants) and peri-implantitis. In observations over a period of 5–11 years it has been reported that peri-implant mucositis affects 40–90% of implants in 80% of subjects, and that approximately 20% of implants develop peri-implantitis, which can lead to bone resorption and eventually implant loss [17].

Microbiological studies conducted on healthy peri-implant tissues have demonstrated the presence of high proportions of coccoid cells, with a low proportion of anaerobic and aerobic species, a small number of Gram-negative species, and a low evidence of periodontal pathogenic bacteria [18,19,20]. Gram-positive aerobic bacteria such as *Streptococcus mitis*, *Streptococcus sanguis* and *Streptococcus oralis* have been observed on the surfaces of dental implants surrounded by a healthy oral environment but in low concentrations and anaerobic Gram-negative bacilli [21].

The general conclusion, that the common etiology of peri-implant pathogens is the microbial colonization of implant surfaces, has oriented research in the field of biomaterials intended for the manufacture of dental implants, towards the promotion of antibiotic materials loaded with antibacterial substances, or coated with anti-adhesive/antibacterial agents [22,23].

The idea of coating titanium or titanium alloy dental implants with Ag in order to prevent local and systemic infections in the implantation process is based on the microbial inhibition produced by Ag and proven since 1893 [24].

Ideally, an implant should have a sufficient amount of Ag to be antimicrobial for a few days after surgery, to help minimize the risk of postoperative infection, but also to promote osseointegration.

The mechanisms by which silver inhibits the growth of bacteria are determined by the way it is administered. From this point of view, salts such as silver nitrate (AgNO_3_) have proven their effectiveness in antibacterial activity by continuously releasing a moderate amount of silver ions. In its highly reactive ionized form, silver binds to proteins in the bacterial cell envelope and brings structural changes, both in the cell wall and in the cytoplasmic membrane [25,26,27,28,29].

The efficacy of silver particles in antibacterial coatings based on the control of the size and shape of the particles (nanoparticles) is explained by the large specific surface area and implicitly by a better physical contact with the cell wall of the bacterium, thus producing the deactivation of cellular enzymes and the disruption of membrane permeability [30]. Recent studies admit that a high concentration of small Ag particles (nanoparticles) can cause apoptosis in human cells, the mechanism of cytotoxicity being related to their nano-size. The toxicity of silver nanoparticles at a high concentration can most likely be attributed to their increased surface area and the release of ions that bind to proteins and nucleic acids interfering with their functions [31,32,33].

The identification of the potential adverse effects of the use of silver is an aspect that needs to be clarified, and the clarification of the mechanism of cytotoxicity, an issue considered as important as research on the applications and risk assessment of the use of silver in antibacterial protection [34]. However, this requires standard toxicology tests, a standardized format of clinical utility, which focuses on cytotoxicity and the lowest dose necessary to achieve the best therapeutic effect [35,36,37,38].

In most studies investigating cytotoxicity using MTT assays and osteogenic differentiation by measuring activity, convenient cytotoxicity was observed when silver nanoparticles were added to the titanium surface using a polymer as a surface coating and/or when a micro/nanostructured surface such as nanotubes was used. Some research laboratories are focusing on developing methods to reduce the toxicity of nanoparticles.

One solution was to combine silver with other polymeric and ceramic substances. Yu et al. developed hybrid lysozyme/chitosan/silver/HA coating materials on the titanium surface and demonstrated that the toxic properties of silver were reduced by chitosan, without affecting the antibacterial properties and stability of silver nanoparticles [39]. It is also recognized that when hydroxyapatite (HAP) is combined with silver nanoparticles, antimicrobial properties can also be improved at the same time; the Ag-HAP system inhibits microbial infection and improves bone tissue formation [40,41,42,43,44].

However, as pointed out in some studies [45,46,47,48], the addition of silver nanoparticles to provide antibacterial properties to the dental implant, could, at the same time, undermine its biocompatibility with human tissue; biocompatibility with osteoblasts that are essential for osseointegration of the implant into the surrounding bone is very important, although the precise concentrations of Ag nanoparticles that are biocompatible with osteoblasts are poorly understood. Studies on the cytotoxic effects and biocompatibility of silver nanoparticles, especially in vivo animal studies, are still limited, and studies on systemic adverse effects of silver nanoparticles on dental implants have not been clinically conducted.

The research carried out in the present study focuses on evaluating the effect produced by hydroxyapatite, when it is co-present in the antibacterial coating with silver. By measuring the viability of human osteoblasts, the contribution of hydroxyapatite in reducing the cellular activity produced by silver is comparatively assessed and thus highlighted. Although silver-based surfaces are widely investigated for infection prevention, their therapeutic window is constrained by dose-dependent cytotoxicity toward osteogenic cells. Accordingly, combining Ag with a bioactive calcium phosphate phase may offer a strategy to improve cytocompatibility while preserving the antimicrobial rationale reported in prior studies.

## 2. Materials and Methods

### 2.1. Substrate Materials

**Figure 1:**
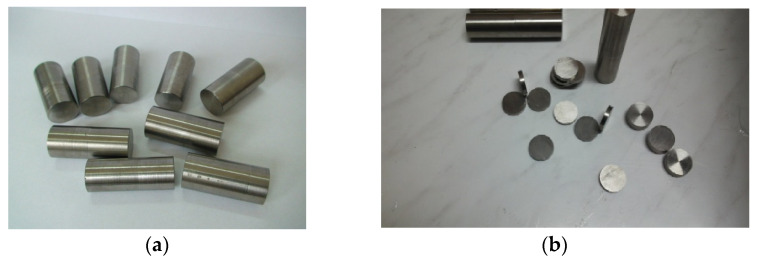
(**a**). Final semi-finished product resulting from casting, (**b**). Disk samples with a diameter of 10 mm and a thickness of 3 mm, cut from cast semi-finished products.

Chemical deposits were made on disk samples with a diameter of 10 mm and a thickness of 3 mm, taken from the binary bioalloy TiZr obtained by casting (Figure 1a,b).

### 2.2. Coating Procedure

For the chemical coating with metallic silver, solutions prepared with chemically pure reagents and double-distilled water were used: 100 mL of 2% AgNO_3_ were treated with 50 mL of 5% NaOH. In the container with the prepared solution (heated to approximately 50 °C), the samples (3–5 samples/regime) were introduced and maintained for approximately 10 min together with a magnetic stirrer to ensure homogenization of the system. Under stirring at 500 rpm, 2 mL of formaldehyde 30% solution ware added. Subsequently, the samples were washed with distilled water and placed in a drying oven at 105 °C.

Chemical reactions involved in the deposition of metallic silver:

(1) $$\left({\mathrm{Ag}}^{+}+{\mathrm{NO}}^{3-})+({\mathrm{Na}}^{+}+{\mathrm{HO}}^{-})=\mathrm{Ag}(\mathrm{OH})\;(\mathrm{white}\;\mathrm{precipitate})↓+\;({\mathrm{Na}}^{+}+{\mathrm{NO}}^{3-}\right)$$

(2) $$2\mathrm{AgOH}\to {\mathrm{Ag}}_{2}O\;(\mathrm{black}\;\mathrm{precipitate})↓+\;H_{2}O$$

Ag_2_O + NH_3_ + H_2_O → ([Ag(NH_3_)_2_] + HO^−^) (colorless complex combination) (3)

(4) $$H-C=O–H+2\;([\mathrm{Ag}({\mathrm{NH}}_{3}{)}_{2}]+{\mathrm{HO}}^{-})\to 2\;\mathrm{Ag}+H\text{-}C_{\backslash OH}^{⸗O}+4{NH}_{3}$$

Both the chemical reactions and the conditions for the deposition of metallic silver on TiZr samples are described in detail in previously published studies [49,50,51]. Next, hydroxyapatite was applied to these samples. The synthesis of hydroxyapatite was achieved by chemical coprecipitation using calcium hydroxide and orthophosphoric acid as precursors. A prepared solution of 0.2 mol/L orthophosphoric acid was added, by dripping, over the suspension of 4% calcium hydroxide in distilled water, intensely stirred. The equivalence pH was measured and the maturation of the hydroxyapatite thus obtained was continued for 1 h.

### 2.3. Surface Characterization (SEM/EDS, XRD, FT-IR)

The characterization of the deposition was achieved by studying the morphology of the sample surface, qualitative and quantitative compositional analysis by electron microscopy with SEM electron scanning equipped with an EDS analyzer and software autocalibration on a standardless method. Scanning electron microscopy was performed with the Thermo Fisher Quattro S equipment (Thermo Fisher Scientific, Waltham, MA, USA). The investigations on the deposited layer have been extended to include XRD analysis performed using a Shimadzu XRD 6000 diffractometer (Shimadzu, Kyoto, Japan), equipped with Ni-filtered CuKα radiation (λ = 1.5406 Å) and FT-IR analysis using a Nicolet IS50 FTIR spectrometer (Thermo Fisher Scientific, Waltham, MA, USA) equipped with a built-in ATR accessory, DTGS detector, and KBr beam splitter.

A total of 40 scans were co-added over the range of 4000–400 cm^−1^ with a resolution of 4 cm^−1^. The air was taken as a reference for the background spectrum before each sample. After each spectrum, the ATR plate was cleaned with ethanol solution. In order to verify that no residue from the previous sample remained, a background spectrum was collected each time and compared to the previous background spectrum. The FT-IR spectrometer was sited in a room that was air conditioned with controlled temperature (21 °C).

### 2.4. Biological Testing

#### 2.4.1. Cell Viability Assay (MTT)

Human osteoblasts (hFOB 1.19-CRL-11372 cell line, acquired from ATCC, Manassas, VA, USA) were used for in vitro testing of cell viability and adhesion. They were maintained at a temperature of 34 °C in a humidified atmosphere with 5% CO_2_ in Dulbecco modified Eagle (DMEM)/Ham’s F-12 medium without phenol red (1:1; Sigma-Aldrich, Darmstadt, Germany), supplemented with 10% fetal bovine serum (FBS), 2.5 mM of L-glutamine (Sigma) and 0.3 mg/mL of antibiotic G418 (Sigma). The cells were seeded at a density of 2 × 10^4^ cells/cm^2^ on the surface of the tested samples, previously sterilized for 2 h under UV light, and on the plastic surface of the 6-well tissue culture plate, which served as a control of the experiments. The biocompatibility tests were performed after 8 days of incubation under standard conditions, according to the full description by Vasilescu and collaborators [52]. The MTT test was used to assess cell viability by measuring mitochondrial activity. After the end of exposure, the cells were incubated with a 1 mg/mL MTT solution for 3 h at 37 °C. The formed formazan crystals were dissolved in isopropanol, and absorbance was measured at 595 nm using a microplate reader. Cell viability was expressed as a percentage relative to control.

#### 2.4.2. Fluorescence-Based Viability and Adhesion Assay

Calcein-AM and ethidium homodimer-1 solutions from the LIVE/DEAD™ Viability/Cytotoxicity Kit (Thermo Fischer, Waltham, MA, USA) were used to label live and dead cells, respectively, according to the manufacturer’s instructions. After 30 min of incubation, cells were visualized using an Olympus IX71 fluorescence microscope (Olympus, Tokyo, Japan).

Morphological changes in the actin cytoskeleton were highlighted using phalloidin conjugated with FITC. The culture medium was removed and the cells were fixed with 100 µL/well of 4% paraformaldehyde in PBS for 20 min at room temperature. Cell membranes were permeabilized with 0.1% Triton X-100-1.2% BSA in PBS for one hour, followed by three washes with PBS. The cells were then incubated for one hour with 130 µL phalloidin–FITC solution (20 µg/mL) to stain actin filaments, while nuclei were labeled with DAPI (2 µg/mL) for 15 min. After three additional PBS washes, the cells were visualized using an Olympus IX71 inverted fluorescence microscope (Olympus, Tokyo, Japan).

### 2.5. Statistical Analysis

The results obtained on human cells were represented as the mean value ± standard deviation (SD) from three different experiments. The statistical analysis was performed using comparisons between groups evaluated by unidirectional ANOVA, followed by the Bonferroni post hoc test (GraphPad Prism software, version 5; GraphPad Software, Inc., La Jolla, CA, USA). A *p* value less than 0.05 was considered statistically significant.

## 3. Results

### 3.1. Results Obtained from Surface Analysis of Samples (SEM/EDS)

**Figure 2:**
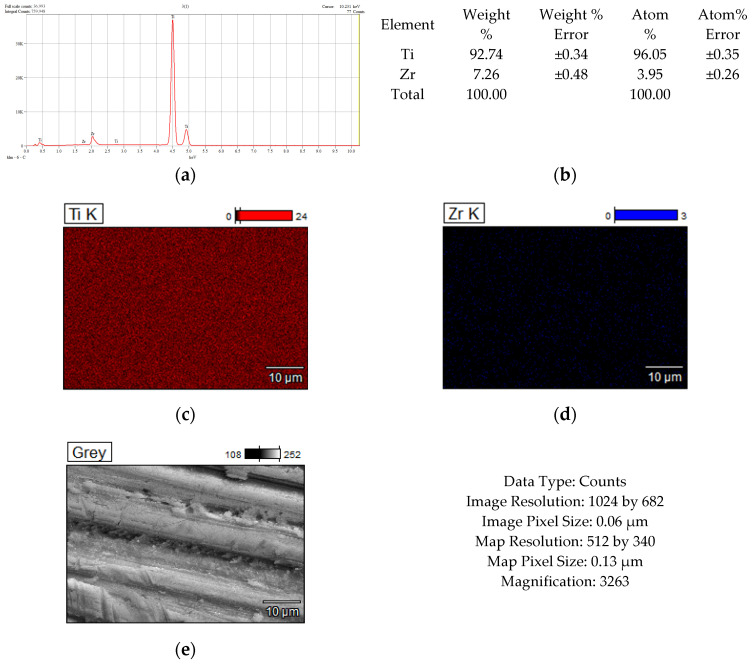
Chemical composition analysis results corresponding to TiZr support: (**a**) Energy Dispersive Spectra; (**b**) EDS semiquantitative results; (**c**,**d**) mapping elemental distribution; (**e**) SEM microscopical surface aspect.

**Figure 3:**
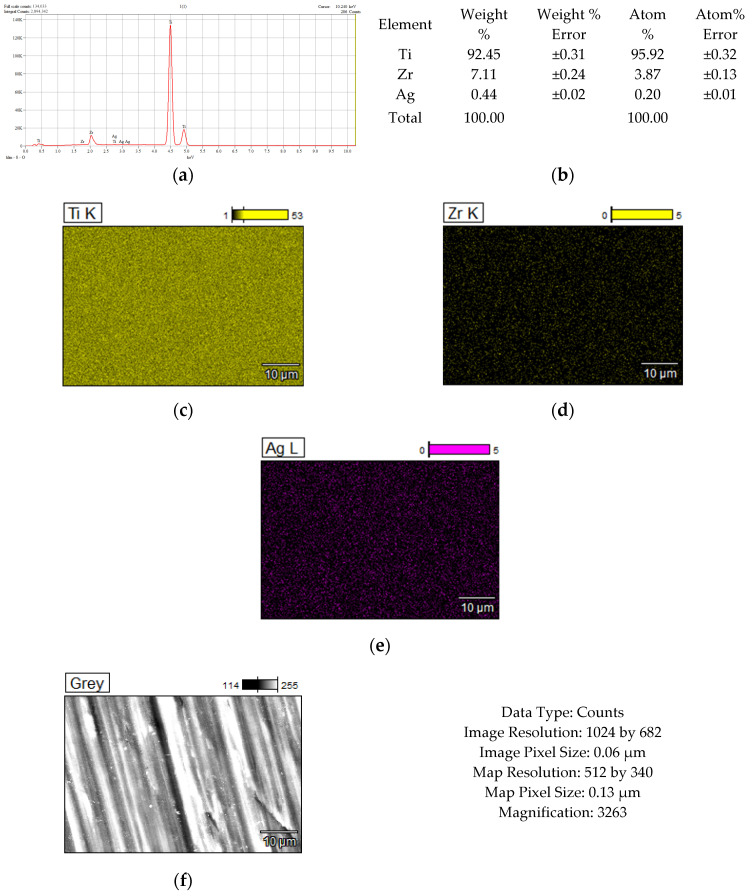
Chemical composition analysis results corresponding to TiZr support with Ag deposition: (**a**) Energy Dispersive Spectra; (**b**) EDS semiquantitative results; (**c**–**e**) mapping elemental distribution; (**f**) SEM microscopical surface aspect.

**Figure 4:**
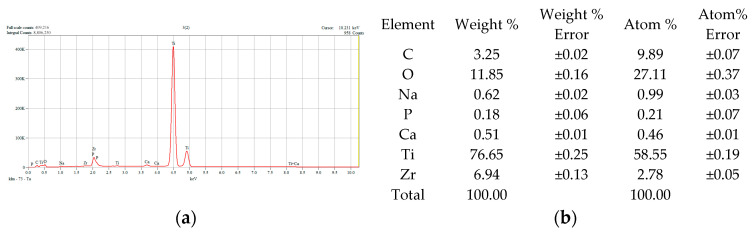

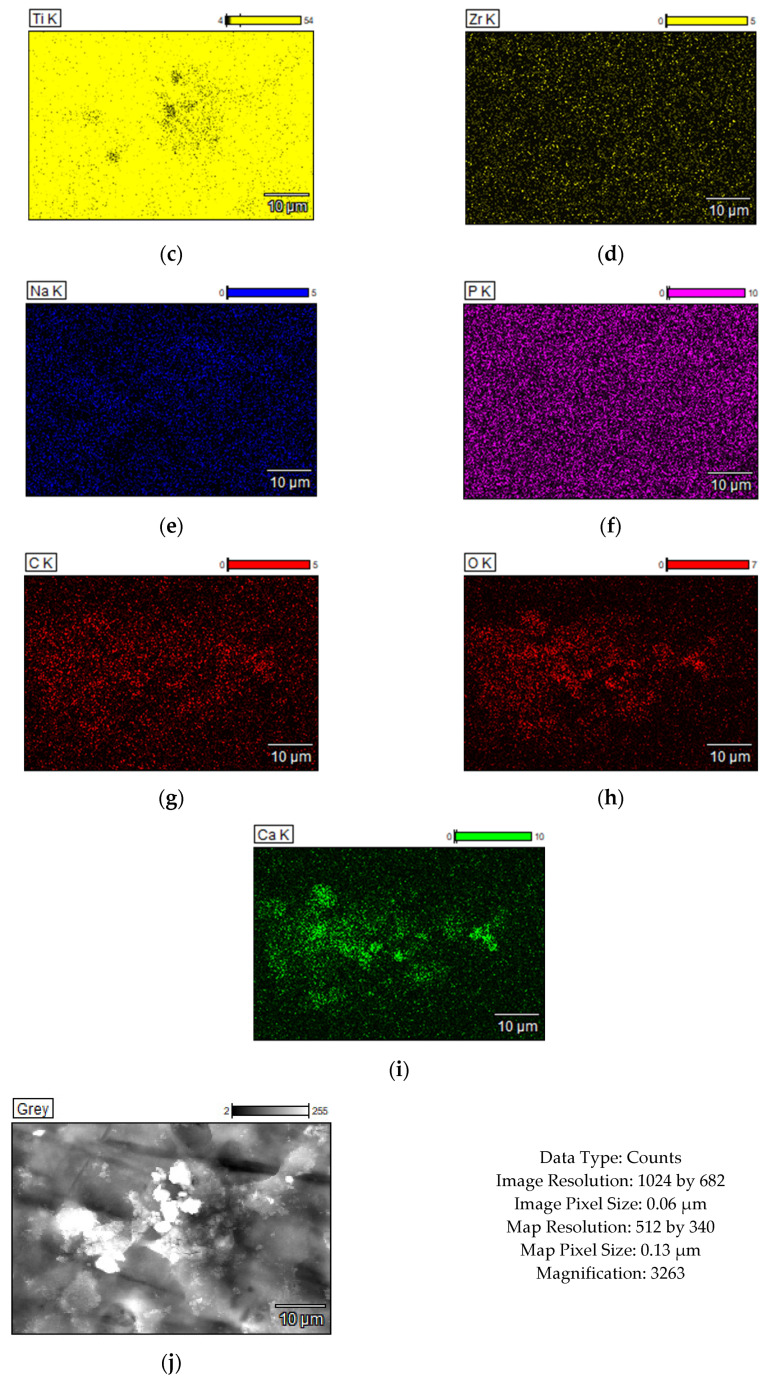
Chemical composition analysis results corresponding to TiZr support with HAP deposition: (**a**) Energy Dispersive Spectra; (**b**) EDS semiquantitative results; (**c**–**i**) mapping elemental distribution; (**j**) SEM microscopical surface aspect.

**Figure 5:**
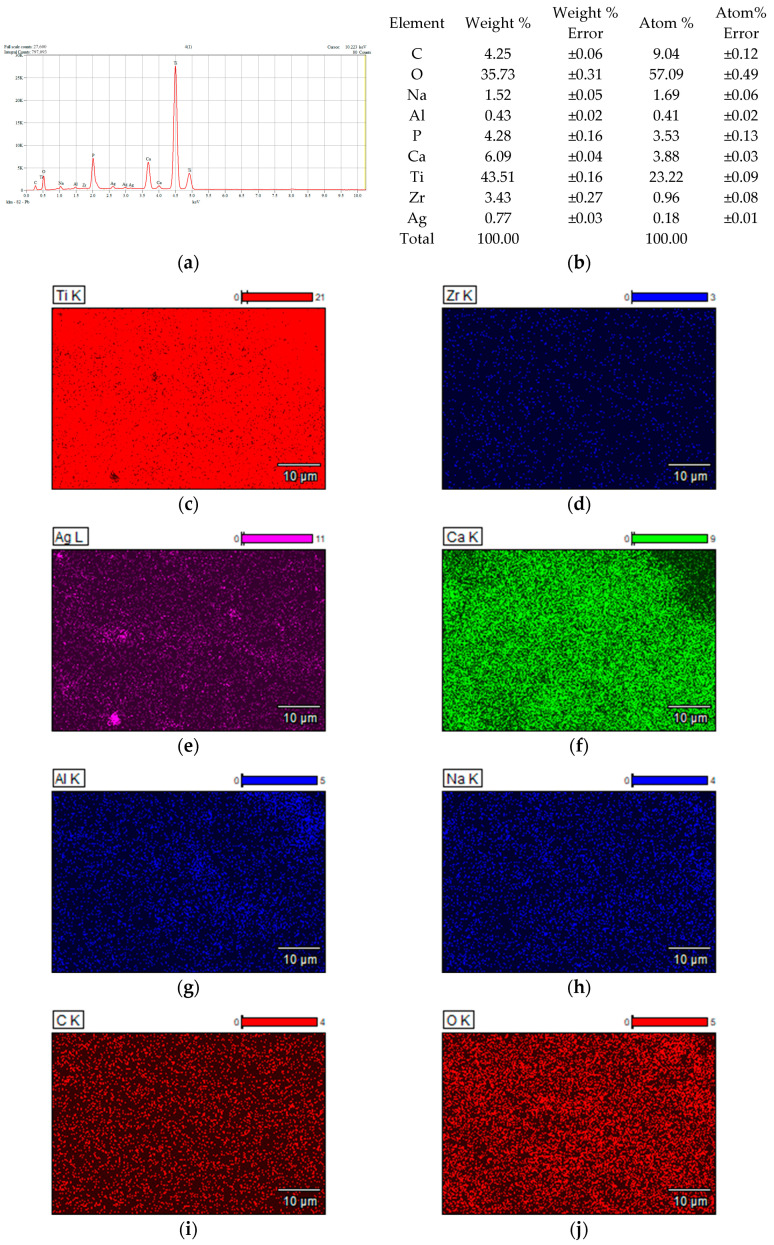

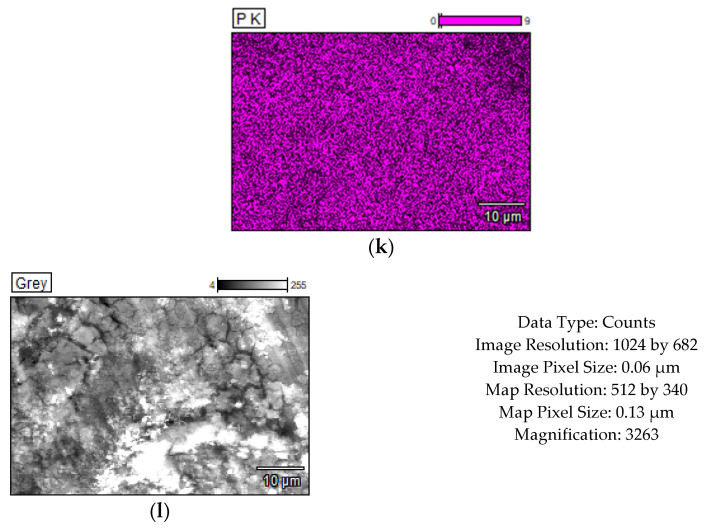
Chemical composition analysis results corresponding to TiZr support alloy with HAP+ Ag deposition: (**a**) Energy Dispersive Spectra; (**b**) EDS semiquantitative results; (**c**–**k**) mapping elemental distribution; (**l**) SEM microscopical surface aspect.

Figure 2, Figure 3, Figure 4 and Figure 5 show the results of the compositional analysis and SEM aspects of the experimental samples.

In the images obtained through Scanning Electron Microscopy (SEM), the presence of silver particles is noticeable, small precipitates with diameters ranging from 3 to 5 microns, characterized by a high degree of dispersion (Figure 3f). During HAP deposition, the covered material islands have a larger diameter, approximately 100 microns, with a compact appearance (Figure 4j). In the case of samples subjected to Ag + HAP deposition (Figure 5l), a hybrid coating material appearance is evident, characterized by a heterogeneous network of HAP incorporating small silver precipitate particles.

### 3.2. XRD Analysis

**Figure 6:**
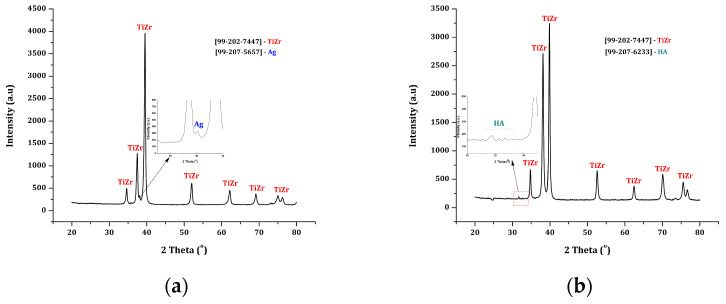
XRD diffractograms recorded for the experimental samples: (**a**). Ag deposition; (**b**). HAP deposition.

Figure 6 displays the results of the XRD diffractogram recorded for the experimental samples.

XRD analysis confirms the stability of the TiZr structure, highlighting diffraction peaks and intensities that correspond to the ASTM sheet for the TiZr phase [99-202-7447], as well as the presence of silver on the surface (peak at 38° (2θ), attributed to metallic silver, in accordance with the ASTM sheet [99-207-5657]), and hydroxyapatite (HAP), with peaks in the range of ~31–33° (2θ), characteristic positions of the crystalline phase of hydroxyapatite, according to the ASTM sheet [99-207-6233]. The reduced intensity and broadening of these maxima indicate the presence of a thin layer of HA, characterized by common features observed in fine ceramic coatings applied to metallic substrates.

### 3.3. FT-IR Analysis

**Figure 7:**
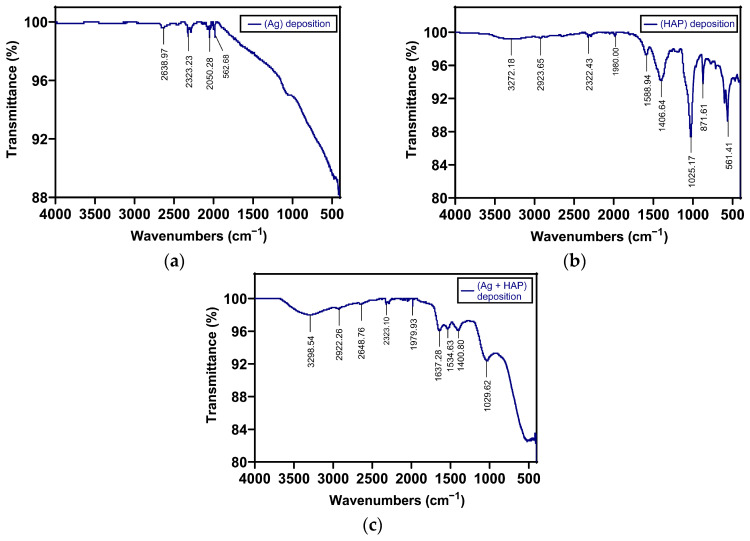
FT-IR analysis of TiZr-based sample materials with (**a**) Ag deposition, (**b**) HAP deposition, (**c**) Ag + HAP deposition.

FT-IR analysis confirmed the chemical deposition of Ag on TiZr through the presence of a specific band in the 2000–400 cm^−1^ region, with a reduction in baseline transmittance observed due to the presence of Ag (Figure 7a,c).

In the case of samples with hydroxyapatite (HAP) deposition, the chemical deposition on TiZr was also confirmed (Figure 7b,c). Hydroxyapatite molecules contain phosphate and hydroxyl groups in their chemical structure, which have active vibrations in the infrared spectrum. The PO_4_^3−^ and OH^−1^ groups generate vibrational bands at different wavenumbers (Figure 7b). Stretching modes were observed at wavenumbers of 962 and 1029 cm^−1^, while bending vibrations produced weak bands at 562 and 602 cm^−1^. The band at 1029 cm^−1^ can be attributed to asymmetric stretching vibrations, and the band at 962 cm^−1^ is due to symmetric stretching vibrations of the phosphate group. The broad band from 3300 to 3550 cm^−1^ can be attributed to the vibrational stretching of the O-H group. Similar bands have been found for hydroxyapatite in the other published literature [53,54,55,56,57]. These bands, attributed to the phosphate and hydroxyl groups in the chemical structure of HA, were identified in the infrared spectrum of the hydroxyapatite deposition sample (HAP), confirming chemical deposition on the TiZr-based material (Figure 7b,c). The hydroxyl group produced a band in the 3500–3300 cm^−1^ range, while the bands attributed to the phosphate group were observed at 960 cm^−1^, 1025 cm^−1^, 600 cm^−1^, and 561 cm^−1^ (Figure 7b). For samples with Ag + HAP deposition, the chemical deposition of Ag on the Ti-based material resulted in fewer bands in the same range, mainly bands at 1029 cm^−1^ and 3500–3300 cm^−1^ (Figure 7c). The FT-IR results demonstrated the presence of chemical deposition of HA and Ag on Ti-based materials (Figure 7).

### 3.4. Assessment of Cell Response

**Figure 8:**
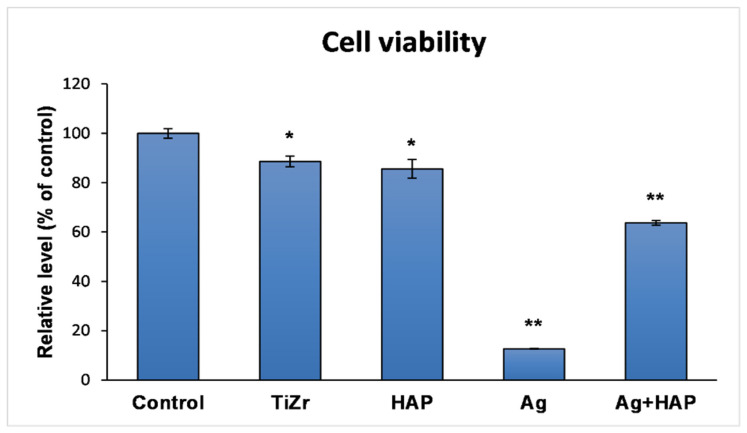
Viability of human fetal osteoblasts (hFOB cell line) after 8 days of incubation on the surface of uncoated and coated TiZr samples. The results are calculated as mean ± standard deviation from three independent experiments and are presented relative to the control—the plastic surface of the plate with 96 wells; * *p* < 0.05 and ** *p* < 0.01 compared to control.

MTT assay (Figure 8) performed after 8 days of incubation revealed that human osteoblast viability was 88.58 ± 2.16% for the uncoated TiZr support (*p* < 0.05 vs. control). In contrast, viability significantly decreased to 12.65 ± 0.16% for Ag-coated samples (*p* < 0.01 vs. control). The TiZr-Ag-HAP coating significantly improved cell viability to 63.69 ± 0.92% (*p* < 0.01 vs. control). The measurement of the viability of human osteoblasts showed a significant decrease in cellular activity following exposure to silver deposited on the TiZr alloy, indicating a pronounced cytotoxicity associated with the presence of this antimicrobial agent (Figure 8). However, when hydroxyapatite is co-present in the system, the cytotoxic effect of silver diminishes considerably, suggesting a protective role of hydroxyapatite on osteoblasts.

**Figure 9:**
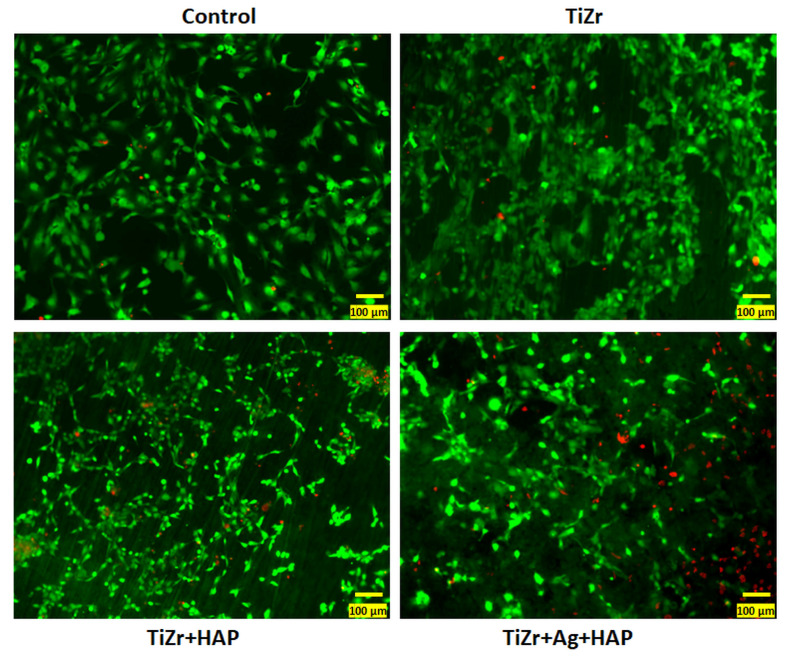
Fluorescent staining of live cells (green labeling with calceine solution) and dead cells (red labeling with propidium iodide) after exposure of human osteoblasts to TiZr surfaces coated with Ag and HAP. All images were obtained with the 20× objective, scale bar: 50 μm.

Therefore, the TiZr plates covered with HAP and HAP + Ag were further studied by Live/Dead viability test. The fluorescence microscopy images (Figure 9) were consistent with the results regarding cell viability.

**Figure 10:**
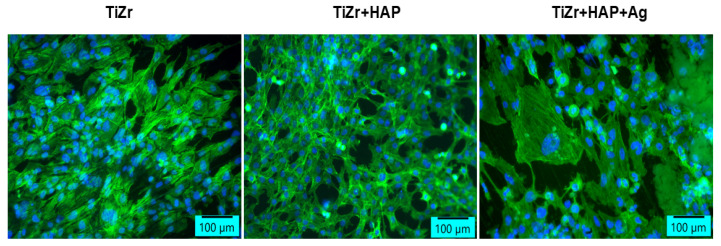
Fluorescent labeling of actin filaments in human osteoblasts after 8 days of incubation with Ag- and HAP-coated samples (green: F-actin labeled with phalloidine-fluorescein isothiocyanate; blue: nuclei labeled with 4′,6-diamidino-2-phenylindole; scale bar: 100 μm).

Also, the labeling of actin filaments (Figure 10) showed a higher adhesion in HAP compared to HAP + Ag coverage, but the morphology and behavior of human osteoblasts were not disturbed in the presence of these coated surfaces. Both types of deposits stimulate the adhesion of osteoblasts, which are organized in compact groups of cells, distributed over the entire surface of the sample.

## 4. Discussion

Silver coatings are known for their antibacterial properties. In implantology, they help prevent infections (local and systemic), which can occur as a result of bacterial contamination. The mechanisms by which silver inhibits bacterial growth depend on how it is administered. In this regard, silver salts such as silver nitrate (AgNO_3_) have proven effective in antibacterial activity through the continuous release of a moderate amount of silver ions. In its highly reactive ionized form, silver binds to proteins in the bacterial cell wall and causes structural changes, both in the cell wall and in the cytoplasmic membrane [58].

In a previous published study [59], I analyzed the antibacterial efficacy of silver and silver in the presence of chitosan by measuring the diameter of the bacterial inhibition zone in relation to the bacterial strains *Escherichia coli* and *Staphylococcus aureus*. The results indicated aspects related to the resistance difference between the two bacterial strains, attributed to differences in their cell wall structures. The study also revealed the influence of the silver concentration in the layer and the effect of chitosan on the diameter of the inhibition zones. The antimicrobial potential and effectiveness of silver particles in antibacterial coatings depend significantly on the concentration, size, and shape of the particles. Small-sized particles (nanoparticles), due to their large specific surface area for better physical contact with the bacterial cell wall, deactivate cellular enzymes and disrupt membrane permeability [60].

Analyzing the bactericidal effect of silver nanoparticles with sizes ranging from 1 to 100 nm, it appears that nanoparticles that have a direct interaction with bacteria preferably have a diameter of approximately 1–10 nm; at these sizes, the nanoparticles attach to the surface of the cell membrane and significantly disrupt its functions, such as respiration and permeability [61]. Other studies indicate that 25 nm nanoparticles exhibit the greatest antibacterial activity and are toxic to bacterial cells at concentrations below 1.69 μg/mL of Ag [62].

The toxicity of silver nanoparticles can most likely be attributed to their increased surface area and the release of ions that bind to proteins and nucleic acids, interfering with their functions [63]. Silver nanoparticles are toxic to both microorganisms and human cells, which may be a disadvantage in interactions with biological systems.

The study conducted by Besinis et al. presents intriguing observations, indicating that a coating of silver nanoparticles on titanium dental implants may possess antibacterial properties against *Streptococcus sanguinis* both in vitro and in the prevention of biofilm formation. However, they also noted that the incorporation of silver nanoparticles could potentially compromise the biocompatibility of the implants with human tissue [43].

Therefore, a significant portion of contemporary research endeavors to develop methodologies for mitigating nanoparticle toxicity. One approach involves the combination of silver with other polymeric and ceramic substances [31,35].

Yu and colleagues developed hybrid coating materials based on lysozyme, chitosan, silver, and HA on titanium surfaces, demonstrating that the toxic properties of silver were reduced through the use of chitosan without affecting the antibacterial properties and stability of silver nanoparticles [39].

Our research dedicated to analyzing the influence of hydroxyapatite on the viability and morphology of human osteoblasts provides useful data that complement the information about the role of bioactive components in combination with silver used in antibacterial coatings. Despite all limitations, the study reveals a link between the effect of hydroxyapatite and the potential toxicity of silver, expressed through decreased cell viability observed in experiments. In the absence of direct Ag release measurements, the presented data support an association between Ag-based surface functionalization and reduced osteoblast viability.

Measuring the viability of human osteoblasts showed a significant decrease in cellular activity after exposure to silver deposited on the TiZr support, but this reduction is counterbalanced by the presence of hydroxyapatite in the system, suggesting a protective role of hydroxyapatite on osteoblasts.

In the case of the support (TiZr), cell viability was 80% (* *p* < 0.05), and for the silver-coated metal support, there was a significant decrease below 20% (*p* < 0.05). In the presence of hydroxyapatite, cell viability improved, remaining at an acceptable level of 60%.

Hydroxyapatite (HAP) is recognized as an agent that enhances biocompatibility due to its bioactive and osteoconductive nature, as well as its chemical composition similar to the mineral matrix of bone tissue. In vitro studies also show that HAPs can reduce oxidative stress and promote cell proliferation, even in composites with metals or polymers [64,65]. Although a thin layer as indicated by XRD analysis, the presence of HAP led to an increase in cell viability above the threshold, which can be considered biologically relevant, especially in the context of a significant increase compared to the TiZr support coated solely with Ag.

The significant reduction in viability observed on surfaces containing only Ag and the improved response on Ag + HAP surfaces indicate that incorporating a calcium phosphate phase can mitigate this negative cellular response and may enhance the effectiveness of silver in preventing infections.

### Limits of the Study

The study contains observations and useful data regarding the formation of coating layers made through simple, inexpensive, accessible methods such as chemical deposition. It also highlighted a way to reduce the potential adverse effects of Ag in antibacterial coatings. However, the study has limitations related to the need for further investigations for a detailed analysis of the coverage characteristics, such as thickness, uniformity, and interfacial adhesion. Although the presence of coating elements in the deposited layer is confirmed through XRD and FT-IR techniques, research for complete characterization and testing of the layer’s properties is ongoing in our subsequent studies.

The biological evaluation was limited to in vitro criteria, such as the MTT test, live/dead cell staining, and actin filament labeling, using a single osteoblast cell line (hFOB 1.19) after 8 days of incubation. Although these results provide consistent evidence that co-deposition of hydroxyapatite mitigates the significant decrease in viability observed for coatings with only Ag, additional tests and more advanced conditions in the testing process would help strengthen the findings. Although it provides useful information, I consider that the study results need to be supported further by additional tests and more advanced testing conditions, which would help strengthen the findings.

The potential adverse effects of Ag in antibacterial coatings must be further demonstrated through experiments based on quantitative measurements of Ag ion release, such as ICP analysis, as well as through dose–response evaluations.

## 5. Conclusions

The results of the study highlighted that the potential cytotoxic effects of silver in antibacterial coatings can be reduced when silver is combined with biocompatibility-enhancing agents, such as hydroxyapatite. Within the limits of the current in vitro model, co-deposition of hydroxyapatite (HAP) mitigated the significant decrease in osteoblast viability observed for coatings with only silver on the TiZr alloy metallic support.

Although limited, the experimental study provides concrete data on chemical coatings with antibacterial efficacy by testing the viability of human osteoblasts under specific experimental conditions; TiZr-Ag-HAP coatings may offer a balance between antimicrobial activity and the biocompatibility necessary for osteointegrative applications by modulating the negative impact of silver ions on human cells. Future research approaches will prioritize obtaining conclusive data by integrating quantitative analysis of Ag release/loading and using standardized antimicrobial tests on the same set of samples to define an optimized performance window.

Their opportunity is justified by the need to clarify aspects regarding the ‘toxicity threshold concentration of silver’ and, if possible, to establish a relevant relationship between antibacterial efficacy and the toxicity limit of silver in antibacterial coatings.

## Data Availability

The original contributions presented in this study are included in the article. Further inquiries can be directed to the corresponding authors.
